# Supplementary material for: Loss of adult skeletal muscle stem cells drives age-related neuromuscular junction degeneration
Source: eLife. 2017 Jun 6;6:e26464. doi: 10.7554/eLife.26464 (PMC5462534; doi:10.7554/eLife.26464)
Supplement: Supplementary file 1. — DOI: http://dx.doi.org/10.7554/eLife.26464.018 [file elife-26464-supp1.docx]

**Supplementary file 1**

qPCR primer list

|  |  | **Sequence (5'->3')** |
| --- | --- | --- |
| Ache | Forward primer | CTCCCTGGTATCCCCTGCATA |
|  | Reverse primer | GGATGCCCAGAAAAGCTGAGA |
| Chrna1 | Forward primer | CTCTCGACTGTTCTCCTGCTG |
|  | Reverse primer | GTAGACCCACGGTGACTTGTA |
| Chrnd | Forward primer | TGAGAAGGGCTACGACAAAGAC |
|  | Reverse primer | GTCTCCTCCACTTCTTTCAGGG |
| Chrne | Forward primer | GAAGCCACTGGAGAGGAACTG |
|  | Reverse primer | AGGGAGATCAGGAACTTGGTTG |
| Etv5 | Forward primer | TCAAGCAGGAATACCATGACCC |
|  | Reverse primer | GGCAGTTAGGCACTTCTGAGTC |
| Musk | Forward primer | GCTGGAAGTGGAGGAAGACAG |
|  | Reverse primer | GTGCAGCGTAGGGTTACAAAG |
| B2M | Forward primer | TTCTGGTGCTTGTCTCACTGA |
|  | Reverse primer | CAGTATGTTCGGCTTCCCATTC |
